# Molecular doping enabled scalable blading of efficient hole-transport-layer-free perovskite solar cells

**DOI:** 10.1038/s41467-018-04028-8

**Published:** 2018-04-24

**Authors:** Wu-Qiang Wu, Qi Wang, Yanjun Fang, Yuchuan Shao, Shi Tang, Yehao Deng, Haidong Lu, Ye Liu, Tao Li, Zhibin Yang, Alexei Gruverman, Jinsong Huang

**Affiliations:** 1 Department of Applied Physical Sciences, University of North Carolina, Chapel Hill, NC 27599 USA; 2 Department of Mechanical and Materials Engineering, University of Nebraska–Lincoln, Lincoln, NE 68588 USA; 3 Department of Physics and Astronomy, University of Nebraska–Lincoln, Lincoln, NE 68588 USA

## Abstract

The efficiencies of perovskite solar cells (PSCs) are now reaching such consistently high levels that scalable manufacturing at low cost is becoming critical. However, this remains challenging due to the expensive hole-transporting materials usually employed, and difficulties associated with the scalable deposition of other functional layers. By simplifying the device architecture, hole-transport-layer-free PSCs with improved photovoltaic performance are fabricated via a scalable doctor-blading process. Molecular doping of halide perovskite films improved the conductivity of the films and their electronic contact with the conductive substrate, resulting in a reduced series resistance. It facilitates the extraction of photoexcited holes from perovskite directly to the conductive substrate. The bladed hole-transport-layer-free PSCs showed a stabilized power conversion efficiency above 20.0%. This work represents a significant step towards the scalable, cost-effective manufacturing of PSCs with both high performance and simple fabrication processes.

## Abstract (web summary)

The existing hole-transporting materials cause problems in the cost and scalability of the perovskite solar cells. Here Wu et al. fabricate high efficiency cells by molecularly doping the perovskite layer without using hole-transporting layers, thus simplify the device architecture and processing steps.

## Introduction

Since their first report in 2009, halide perovskite solar cells (PSCs) have attracted enormous interest as emerging light-to-electricity conversion optoelectronic devices with both high efficiency and ease of solution processability^1–3^. To date, the power conversion efficiencies (PCEs) of solution-processed PSCs have been certified at well above 20%^4–6^. However, all top-performing PSCs were fabricated by a spin-coating method which is unsuitable for high throughput and scalable module production. Several scalable film deposition techniques have been developed for PSC fabrication, such as doctor-blading^7–10^, spray deposition^11^, slot-die coating^12^, solution printing^13^, and electrodeposition^14^. In particular, doctor-blade coating with a controlled substrate temperature has gained most success in depositing high-quality perovskite films with large grains and controllable optoelectronic properties^15^. Moreover, blade coating has great potential to be scaled up for roll-to-roll fabrication. Encouragingly, the PCEs of PSCs with bladed perovskite layers have steadily increased to above 19%, approaching the levels of their spin-coated counterparts^13,16^.

A next step towards the scalable fabrication of PSCs is to coat the charge transport layers by scalable processes. However, the existing multiple-layer device structures, either p–i–n or n–i–p structures with a hole transport layer (HTL), perovskite, and electron transport layer (ETL), impose a great challenge on the scalable fabrication of PSCs in a cost-effective manner^17–20^. First, high-performance organic hole-transporting materials, such as 2,2′,7,7′-Tetrakis[N,N-di(4-methoxyphenyl)amino]-9,9′-spirobifluorene (spiro-OMeTAD), are much more expensive than perovskite materials. Second, for the devices with p–i–n structure, it is difficult to blade-coat perovskite films onto a hydrophobic HTL, e.g., poly(bis(4-phenyl)(2,4,6-trimethylphenyl)amine) (PTAA), while a hydrophilic HTL, e.g., NiO_*x*_, is difficult to form on plastic substrates at low temperature, nor can easily be coated at optimal thickness and electronic quality to achieve the highest device efficiency. Hence, we believe that the elimination of the HTL from the PSC device architecture can be an effective way to solve this challenge. By reducing one step of the standard coating process, at least one third of the manufacture cost and time can be saved, in addition to savings of material cost for the HTL.

The reported ambipolar charge transport characteristics of halide perovskite materials endow them with great potential to construct PSCs with simplified device architecture, such as HTL-free PSCs with either conventional or inverted structure^21–23^. Yet, a critical challenge for HTL-free PSCs is the ineffective hole collection by indium tin oxide (ITO) because of mismatched work functions between ITO and the perovskite, which generally causes significant efficiency loss^23–25^. In addition, the construction of HTL-free devices by the blade-coating method faces more challenges due to rougher and more non-uniform perovskite films, than do those prepared by spin-coating with the anti-solvent method.

Here we report a molecular-doping strategy for a perovskite layer to doctor-blade HTL-free PSCs with a simplified device structure of ITO/perovskite/ETL/Cu. Doping methylammonium lead iodide (MAPbI_3_) films with 2,3,5,6-tetrafluoro-7,7,8,8-tetracyanoquinodimethane (F4TCNQ), a strong electron-withdrawing molecule, leads to a modified ITO/MAPbI_3_ interface with favorable band bending, which facilitates the extraction of photoexcited holes from perovskite to ITO electrode. Combining additive engineering and solvent annealing, high-quality MAPbI_3_ films are obtained with full surface coverage, micrometer-sized grains, and improved crystallinity. By employing a bladed and doped MAPbI_3_ film as the photoactive layer in a HTL-free device structure, a stabilized PCE over 20.0% with almost no *J–V* hysteresis is obtained.

## Results

### Perovskite films fabrication and molecular doping

**Fig. 1:**
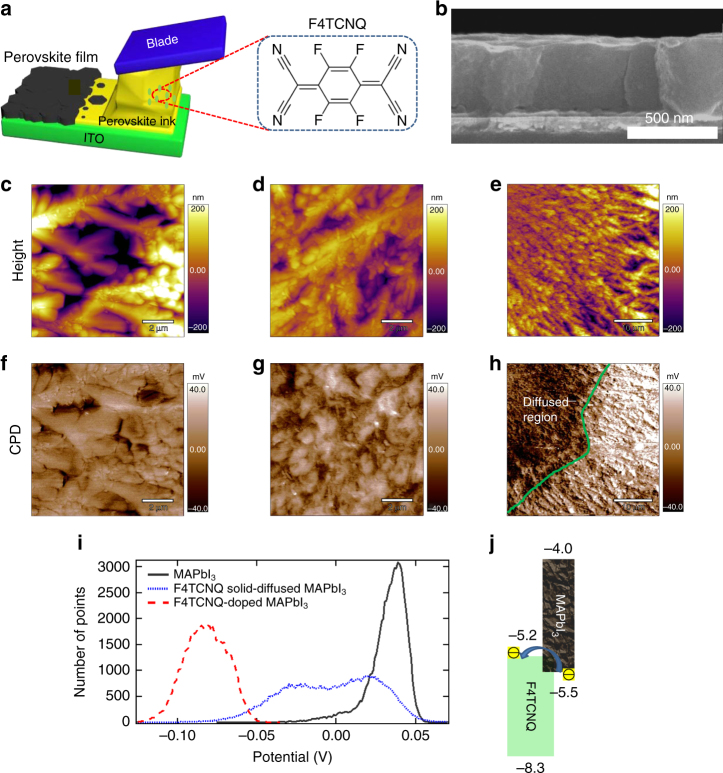
Doctor blading and doping of perovskite films by F4TCNQ. **a** Schematic illustration of a doctor-bladed perovskite film and the chemical structure of F4TCNQ dopant. **b** Cross-sectional SEM image of the MAPbI_3_ film deposited directly onto ITO glass via bladed coating at 150 °C, showing the film thickness of around 500 nm. Topography KPFM images of **c**, **f** MAPbI_3_, **d**, **g** F4TCNQ-doped MAPbI_3_, and **e**, **h** F4TCNQ solid-diffused MAPbI_3_ films. CPD represents the contact potential difference between the tip and sample’s surface. **i** Surface potential profiles of different perovskite films as indicated. **j** Schematic illustration of the energy diagram and electron transfer process for MAPbI_3_:F4TCNQ blends

The MAPbI_3_ perovskite films were directly deposited onto blank ITO glass substrates by a previously reported doctor-blading method^9^, which is schematically illustrated in Fig. 1a. Before blade coating, the ITO substrates were cleaned by a UV-ozone treatment to improve their wettability to perovskite solution^23^. MAPbI_3_ precursor dissolved in N,N-diethylformamide (DMF) containing p-type dopant (F4TCNQ) was dripped onto substrates which were heated at 150 °C, followed by a quick blading process to spread the solution onto the substrates. After blading, the perovskite precursor solution dried in a few seconds, and the substrates were quickly removed from the hot plate and annealed at 100 °C for varied durations. The bladed perovskite films showed thicknesses of 500 ± 20 nm from cross-sectional imaging (Fig. 1b) by scanning electron microscopy (SEM). To implement molecular doping of the perovskite layer, F4TCNQ with chemical structure shown in Fig. 1a was dissolved in DMF separately, and then added to the as-prepared MAPbI_3_ precursor solution. Perovskite films made without or with F4TCNQ were denoted as MAPbI_3_ or MAPbI_3_:F4TCNQ, respectively.

The doping of perovskite is expected to change the work function of the films. Kelvin probe force microscopy (KPFM) was used to determine the surface potential change of perovskite films upon F4TCNQ mixing. In addition to direct mixing of F4TCNQ and MAPbI_3_ in precursor solution, MAPbI_3_:F4TCNQ was prepared by solid diffusion via heating F4TCNQ powder on MAPbI_3_ and then removing the excess F4TCNQ. The topography and contact potential difference (CPD) images of pristine MAPbI_3_ and MAPbI_3_:F4TCNQ films by either standard solution mixing or solid diffusion are shown in Fig. 1c–h. For better comparison, the three CPD images are flattened and presented in the same color scale, while the histograms of the absolute CPD values of the three types of films are plotted in Fig. 1i. The pure MAPbI_3_ film has coarse grains and uniform CPD distribution (Fig. 1c, f). In contrast, solution mixed MAPbI_3_:F4TCNQ film, in which uniform doping is expected, has fine grains and greater heterogeneity of the CPD distribution (Fig. 1d, g). For the sample with solid-diffused F4TCNQ, some small F4TCNQ particles were placed on top of a MAPbI_3_ film, and the CPD imaging was conducted around the edge (the green color line as indicated in Fig. 1h) to include both neat and doped perovskite regions. Such method leads to a clear edge between diffused and undiffused regions that can be visualized in the CPD image (Fig. 1h), thus the CPD heterogeneity is even higher. The change of absolute CPD caused by F4TCNQ doping is clearly demonstrated in Fig. 1i. A decrease of surface potential or in other words an increase of work function was observed for the MAPbI_3_:F4TCNQ films, irrespective of direct mixing or solid diffusion. The increase of heterogeneity for the MAPbI_3_:F4TCNQ films is also reflected in the broadening of the curves. This confirmed p-type doping of MAPbI_3_ by F4TCNQ. Specifically, after the addition of electron-withdrawing F4TCNQ molecule to the MAPbI_3_, owing to the deep lowest unoccupied molecular orbital (LUMO) level of F4TCNQ, electrons in the valance band of MAPbI_3_ can be thermally activated into the LUMO level of F4TCNQ, which leaves holes in MAPbI_3_ and thus causes its p-doping (Fig. 1j). As a result, the hole concentration in the MAPbI_3_ film increased. The carrier concentration change upon F4TCNQ doping has been evaluated by analyzing the dark capacitance vs. voltage (*C–V*) characteristics of the perovskite devices (Supplementary Fig. 1). Charge density profiles were extracted from the *C–V* curves using Mott–Schottky analysis^26,27^. The carrier concentration in the perovskite layer was calculated to be 0.6 × 10^16^ cm^−3^ in the neat MAPbI_3_ film which increased to 2.4 × 10^16^ cm^−3^ after the addition of 0.03 wt% F4TCNQ. This molecular p-type doping strategy enabled the fabrication of an HTL-free PSC with a simplified device configuration but still effective p–n heterojunction (i.e., p-doped perovskite and n-type ETL), and thus enhanced device performance.

### Electronic properties of doped perovskite films

**Fig. 2:**
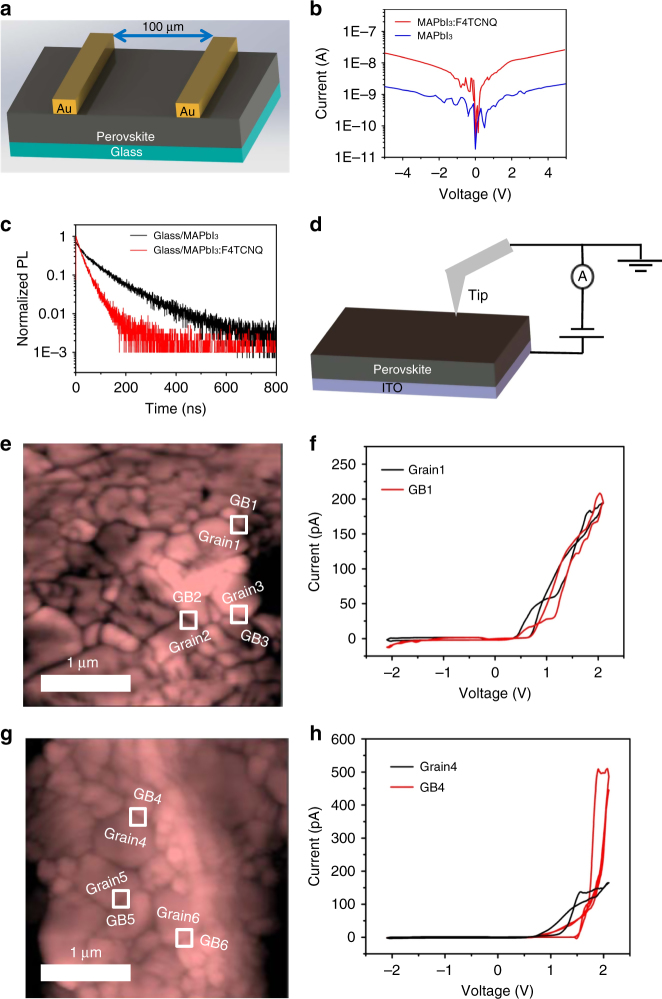
Conductivities and photoluminescence lifetimes of neat or doped perovskite films. **a** Geometry for the lateral conductivity measurement: the perovskite films were 500 nm thick, with 1 mm gold (Au) electrodes separated by 100 μm. **b** *J–V* curves of neat or F4TCNQ-doped MAPbI_3_ deposited on normal glass substrates, where the current was measured along the lateral direction. **c** TRPL decay curves of glass/MAPbI_3_ and glass/MAPbI_3_:F4TCNQ. PL lifetimes were calculated by single exponential fitting. **d** c-AFM setup. Topographic AFM images of **e** MAPbI_3_ and **g** MAPbI_3_:F4TCNQ films, with locations where the c-AFM tip measured the grain and GB currents. Local dark currents measured at the GBs and on the grains for the **f** MAPbI_3_ film and **h** MAPbI_3_:F4TCNQ film

The electronic properties of the MAPbI_3_:F4TCNQ films were investigated by comparing their current density–voltage (*J–V*) characteristics with pristine MAPbI_3_ films deposited on glass substrates in the dark condition. The lateral conductivity was measured across gold electrodes spaced 100 µm apart (Fig. 2a). As shown in Fig. 2b, the MAPbI_3_:F4TCNQ film exhibited a nearly tenfold increase in conductivity over the neat MAPbI_3_ film, which confirmed that F4TCNQ doped MAPbI_3_. The charge transfer from MAPbI_3_ to F4TCNQ is expected to cause quenching of photoluminescence (PL) from the MAPbI_3_ layer, which was measured by using time-resolved photoluminescence (TRPL) decay for MAPbI_3_ films deposited on glass substrates (Fig. 2c). The MAPbI_3_:F4TCNQ prepared on glass substrate showed a PL lifetime of 65 ns, which is fourfold shorter than the PL lifetime of pristine MAPbI_3_ film (268 ns) on glass.

The relative large molecular size of F4TCNQ likely prevents its integration into the lattice of MAPbI_3_. Therefore, we speculate that F4TCNQ molecules most probably remain at the grain boundary (GB) regions of perovskite films and/or perovskite/electrode interfaces. To determine the lateral distribution of F4TCNQ molecules, the local dark conductivity at grains and GBs was measured using conducting atomic force microscopy (c-AFM). Figure 2d illustrates the c-AFM setup, where the microscope tip was positioned at locations randomly chosen from topographic AFM images (Fig. 2e, g). The tip bias was scanned in the range +2.0 to −2.0 V at a rate of 0.14 V s^−1^. For the neat MAPbI_3_ film, the dark currents were similar at the grain and GB regions (Fig. 2f and Supplementary Fig. 2a, b), which is in accordance with previous reporting^28^. In sharp contrast, the dark currents measured at the GB regions of the MAPbI_3_:F4TCNQ film were notably higher (Fig. 2h and Supplementary Figs. 2c, d), elucidating the remarkably improved conductivity at the GB region after molecular doping of MAPbI_3_ by F4TCNQ. At the regions close to GBs, the F4TCNQ molecules and MAPbI_3_ undergo charge transfer, leading to an increase in carrier density in MAPbI_3_ and thus the enhanced dark conductivity^29^.

### Morphologies and crystallinities of perovskite films

**Fig. 3:**
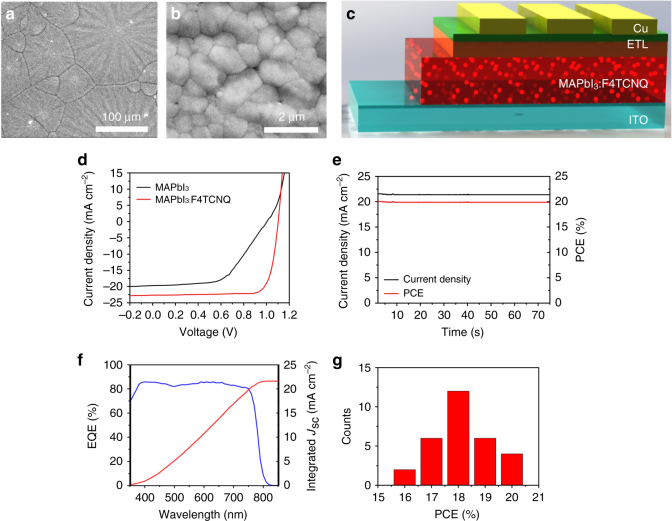
Perovskite film morphology, device structure, and photovoltaic performance. **a** Low magnification and **b** high magnification SEM images of bladed and doped MAPbI_3_ film prepared with MHP (0.225 wt%) and MACl (0.5 wt%) as additive, followed by co-solvents annealing treatment. **c** Schematic illustration of the HTL-free device configuration. **d** *J–V* characteristics, **e** Steady-state current and stabilized PCE measured at a maximum power point (0.93 V), **f** EQE and integrated current density, and **g** PCE histogram of PSCs based on MAPbI_3_:F4TCNQ films (with 0.03 wt% F4TCNQ)

High-quality perovskite films with large grains, high crystallinity and complete surface coverage are also crucial to achieving high-performance HTL-free devices. The blending of F4TCNQ in the perovskite in a small percentage (ranged from 0.01 to 0.05 wt%) did not notably change the morphology and grain size of the perovskite films, as shown by the SEM images in Supplementary Fig. 3, nor cause a drastic change of crystallinity, as shown by the XRD patterns of bladed MAPbI_3_ films prepared with different amounts of F4TCNQ in Supplementary Fig. 4. Accordingly, methylammonium hypophosphite (MHP) and methylammonium chloride (MACl) were introduced as additives to the precursor solution, and co-solvents (dimethyl sulfoxide (DMSO)/ chlorobenzene (CBZ)) post-annealing process was also combined to achieve a high-quality and high-crystalline MAPbI_3_ film (Supplementary Figs. 5, 6 and 7 for SEM images and XRD patterns of bladed MAPbI_3_ films prepared with different amounts of MHP as well as using co-solvents annealing technique in the absence or presence of MACl)^9,10,16,30,31^. Figure 3a, b show representative SEM images of the bladed perovskite films after optimization, which featured a uniform morphology that extended hundreds of micrometers. The perovskite domains were densely packed together, and without observable voids at domain boundaries (Fig. 3a). It should be noted that these domains are not single crystalline grains. Each micrometer-scale convection domain was comprised of micro-sized grains ranging from 800 to 1700 nm (average above 1 μm, Fig. 3b).

### Photovoltaic performances of doped perovskite devices

The impact of molecular doping by F4TCNQ on photovoltaic performance was investigated in detail. As illustrated in Fig. 3c, inverted HTL-free PSCs were fabricated with a device configuration of ITO/MAPbI_3_:F4TCNQ/ETL/Cu. The corresponding photovoltaic performances for PSCs based on different F4TCNQ concentrations have been displayed in the Supplementary Table 1, which indicated the optimal F4TCNQ concentration was 0.03 wt%. Figure 3d shows the current density vs. voltage (*J–V*) characteristics of PSCs based on the pristine MAPbI_3_ films and MAPbI_3_:F4TCNQ films (with 0.03 wt% F4TCNQ), with photovoltaic parameters summarized in Table 1. The reference PSC without F4TCNQ (i.e., MAPbI_3_) exhibited a poor PCE of just 11.0%, which is in accordance with that of most reported HTL-free PSCs^23,24^. Surprisingly, with the addition of F4TCNQ, notable concurrent enhancements of the *J*_sc_, *V*_oc_, and FF have been achieved. The champion cell based on MAPbI_3_:F4TCNQ film achieved the highest PCE of 20.2%, with a *J*_sc_ of 22.7 mA cm^−2^, a *V*_oc_ of 1.10 V, and a FF of 0.81. To the best of our knowledge, this is the record efficiency for the bladed PSCs in the absence of HTLs. For comparison, the PSC based on the PTAA HTL and undoped MAPbI_3_ layer has been fabricated, which showcases a PCE of 19.5%, along with a *J*_sc_ of 22.1 mA cm^−2^, a *V*_oc_ of 1.12 V, and a FF of 0.79. This result is in good agreement with our previous reports^8,9,16^. It is worth noting that the demonstrated HTL-free device showed similar or even better photovoltaic performance, highlighting the effectiveness of such a doping strategy for constructing high-performance PSCs with not only a simpler structure, but also less deposition steps and lower material cost. The *J–V* curves of MAPbI_3_:F4TCNQ-based device were measured in both reverse and forward scan modes at a scan rate of 0.1 V s^−1^, and almost no hysteresis was observed (Supplementary Fig. 8). Figure 3e shows the stabilized photocurrent density of this device as measured at 0.93 V, giving a stabilized PCE exceeding 20.0% under continuous light soaking. This high stabilized PCE output is among the best record for HTL-free PSCs, and is quite remarkable given that the device is prepared using a scalable fabrication method. Figure 3f shows the external quantum efficiency (EQE) of the champion device. The integrated *J*_sc_ from the EQE measurement agreed well with the *J*_sc_ values measured under AM 1.5 G one sun illumination (Fig. 3d). The fabrication procedure was repeated to confirm the reliability and reproducibility of the results. The PCE histogram was collected from 30 independent cells (Fig. 3g), revealing that more than 73% of the cells had PCEs above 18.0%, and more than 93% had PCEs higher than 17.0% under one sun illumination. A study of the stability for the devices with or without HTL has been conducted. The devices were stored under ambient conditions (where humidity and temperature were about 20% and 25 °C, respectively) without any encapsulation. As compared to the devices with HTL, the HTL-free device exhibits a slightly better stability, which maintained over 92% of its initial PCE after storage for 500 h (Supplementary Fig. 9).

**Table 1:** Photovoltaic parameters of PSCs employing neat or doped perovskite layers under one sun light illumination (AM 1.5 G, 100 mW cm^−2^)

PSCs	*J*_sc_ (mA cm^−2^)	*V*_oc_ (mV)	*η* (%)	Average *η* (%)	FF
MAPbI_3_	19.7	1.00	11.0	9.52 ± 0.72	0.56
MAPbI_3_:F4TCNQ	22.7	1.10	20.2	18.85 ± 0.35	0.81

Data for average PCE (*η*) were calculated from at least 30 devices

**Fig. 4:**
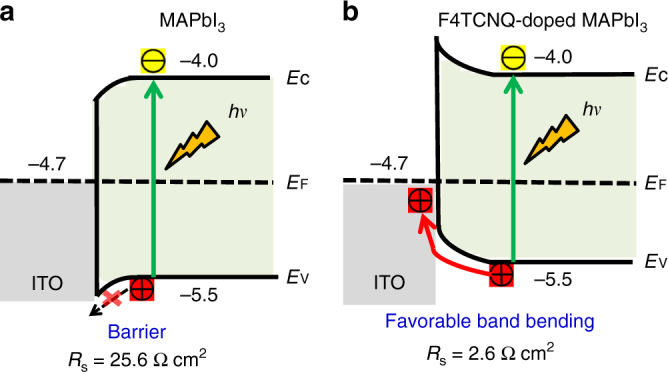
Interfacial hole transfer dynamics. Schematic illustrations of hole transfer at the **a** ITO/MAPbI_3_ or **b** ITO/F4TCNQ-doped MAPbI_3_ interface

It is noted from Fig. 3d and Table 1 that the almost twofold efficiency enhancement in the F4TCNQ-doped devices primarily originates from the remarkable FF increase. Further analysis of the *J–V* curves in Fig. 3d revealed that F4TCNQ doping of the perovskite layer significantly reduced device series resistance (*R*_s_) from 25.6 Ω cm^2^ for undoped device to 2.6 Ω cm^2^ for doped device. The influence of *R*_s_ on device FF has been well established in diode based solar cells^32^, according to the classical equation based on diode equation:

1 $$I = I_{\mathrm{L}} - I_{\mathrm{0}}{\mathrm{exp}}\left[ {\frac{{q(V + IR_{\mathrm{s}})}}{{nkT}}} \right]$$

where *I* is the device output current, *I*_L_ is the maximum light generated current, *I*_0_ is saturated dark current, *R*_s_ is series resistance, *n* is ideal factor, *k* is Boltzmann constant, *T* is temperature. Assuming a diode ideal factor of 1, the FFs for the undoped and doped devices were calculated to be 53% and 83% from Eq. (1), which are very close to measured values of 56% and 81%, respectively. This indicates the increased FF for the devices with doped MAPbI_3_ is mainly caused by the reduction of the series resistance. To find out the origin of reduced series resistance by F4TCNQ doping, energy level diagrams are constructed and shown in Fig. 4 for the perovskite/ITO interface with or without F4TCNQ doping. Here the energy levels were extracted from the literature for the films made with same composition and similar method^33,34^. For undoped MAPbI_3_, the Fermi level of pristine MAPbI_3_ (nearly intrinsic) is slightly lower than that of ITO (−4.7 eV)^34^. The Fermi level lines up during contact formation and causes a downward band-bending in MAPbI_3_, which forms a barrier for hole extraction from MAPbI_3_ to ITO (Fig. 4a). This would also increase the possibility of charge recombination and increase series resistance at the ITO/perovskite interface. In contrast, the incorporation of F4TCNQ in the MAPbI_3_ films turns the MAPbI_3_ to more p-type, particularly at the interface of ITO and MAPbI_3_, which should avert band-bending direction (Fig. 4b). This was confirmed by surface potential measurement that the F4TCNQ doping can increase work function of perovskite by ~0.1 to 0.2 eV (Fig. 1i). As is shown in Fig. 4b, the F4TCNQ doping results in an upward band bending at the ITO/MAPbI_3_:F4TCNQ interface. In this case, the hole transfer from F4TCNQ-doped MAPbI_3_ to ITO is thus facilitated, resulting in a reduced series resistance to increase FF^35^.

The diode Eq. (1) predicts that *R*_s_ variation has no influence on device *V*_oc_, while in our case, the F4TCNQ doping increased device *V*_oc_ from 1.00 to 1.10 V. This discrepancy indicates there is another factor, in addition to *R*_s_, which leads to the *V*_oc_ enhancement. To find out the reason, a transient photovoltage spectroscopy (TPV) measurement was also carried out to understand the charge carrier recombination dynamics. As shown in Supplementary Fig. 10, the MAPbI_3_:F4TCNQ-based PSC had a longer carrier lifetime (0.69 μs) than the reference device based on neat MAPbI_3_ (0.30 μs) under one sun illumination. This result was consistent with the larger *V*_oc_ for the device with doping. The observed carrier recombination lifetime enhancement could be a result of band-bending configuration change due to doping, though there may be other reasons that may also change the carrier recombination lifetime. For instance, for the device with undoped MAPbI_3_, the retarded hole extraction would increase the possibility of charge recombination, because they cannot be effectively extracted out.

## Discussion

We demonstrated how molecular doping influences the series resistance and interfacial charge transfer within HTL-free PSC devices with or without F4TCNQ incorporated in the perovskite layer. In the absence of HTL, both the electrons and holes can transport to the ITO/perovskite interface and recombine there, leading to serious interfacial charge recombination and thus inferior charge collection^36^. In addition, the mismatched work functions between perovskite and ITO would induce an undesirable energy barrier that leads to inferior hole transfer. These situations are all very detrimental to device performance. Molecular doping of perovskite by F4TCNQ to some extent induces favorable interfacial band bending, which allows efficient transport of holes at ITO/perovskite interface (Fig. 4b)^35^.

Choosing an appropriate p-type dopant with proper molecular size and selected surface hydrophility with ITO substrate is promising to further improve the ITO/perovskite interfacial affinity. Owing to the larger molecular size of F4TCNQ than the interstitial sites in perovskite structure, the F4TCNQ concentrated at the grain boundary regions. The dopant assisted with charge dissociation and thus resulting in more balanced transfer of electrons and holes to their respective electrodes, which decreased the possibility of the charge carrier recombination. We found the charge trap density within MAPbI_3_:F4TCNQ-based PSC was even slightly smaller than its pristine MAPbI_3_ counterpart (Supplementary Fig. 11). It has been demonstrated that the trap sites within the organic semiconductors can be reduced due to partial filling by doping, typically at very low doping levels^37^.

In summary, an additive-assisted strategy for p-type molecular doping of solution-bladed perovskite films was demonstrated. F4TCNQ-doped MAPbI_3_ films show increased electrical conductivity, especially at grain boundary regions, and increased charge carrier concentrations. The incorporation of F4TCNQ in perovskite layers could modify the ITO/perovskite interface with reduced series resistance, which could be attributed to the favorable interfacial band bending for facilitated hole transfer and extraction from perovskite to ITO. The simple but effective approach enabled the scalable fabrication of HTL-free PSC devices with a simplified device geometry. Molecular doping of perovskite film by F4TCNQ led to the considerable enhancement of photovoltaic performance from 11.0 to 20.2%. The MAPbI_3_:F4TCNQ-based device exhibited a stabilized PCE that exceeded 20.0% and negligible hysteresis. This effective doping strategy eliminates the HTL preparation step, thus simplifying the PSC fabrication process and reducing costs. Extending the application of this doping technique to a broader range of semiconducting materials will definitely benefit the construction of other high performance, printed optoelectronic devices.

## Methods

### Materials preparation

Lead iodide (PbI_2_, 99.9985%) was purchased from Alfa Aesar. Methylammonium iodide (MAI) was purchased from Dyesol. MACl (98%), 2,3,5,6-tetrafluoro-7,7,8,8-tetracyanoquinodimethane (F4TCNQ, 97%), N,N-dimethylformamide (DMF, anhydrous, 99.8%), DMSO (99.9%), acetonitrile (anhydrous, 99.8%) and CBZ (anhydrous, 99.8%) were purchased from Sigma-Aldrich. All chemicals were used as received without further purification.

### Device fabrication

The ITO glass substrates were ultrasonically washed with deionized water, acetone, and isopropanol for 30 min successively. After drying, the cleaned ITO glass was treated by UV-ozone (UVO) for 15 min then used immediately for device fabrication. To form a perovskite precursor (1 M), an equimolar ratio of PbI_2_ and MAI was dissolved in DMF with different amounts of MHP (0.075 wt% to 0.300 wt%), F4TCNQ (0.01 wt% to 0.05 wt%), and 0.5 wt% MACl (for chlorine-containing precursor) added as required. Specifically, to implement solution-processed molecular doping of the perovskite photoactive layer, F4TCNQ was dissolved in DMF separately, and then added to the solution of as-prepared MAPbI_3_ precursor solution. Typically, MHP/DMF additive was used to increase grain size, improve perovskite film crystallinity, and surface coverage. The bladed coating of perovskite films was conducted in an N_2_-purged glovebox (below 1.0 ppm O_2_ and H_2_O). The perovskite precursor solution (~5 to 10 μL) was dripped onto the ITO glass on a hot plate set at 150 °C, then swiped linearly by a glass blade at a speed of 0.75 cm s^−1^. The as-prepared perovskite films were then annealed at 100 °C for 30 min. For solvent annealing, the perovskite films were annealed under a vapor of mixed DMSO/CBZ solvent (1:1 v/v) at 100 °C for 30 min according to a literature method^31^. Finally, C_60_ (20 nm, Nano-C), bathocuproine (BCP, 8 nm, Sigma-Aldrich), and Cu electrode (80 nm) were sequentially deposited on the perovskite layer by thermal evaporation to form a complete PSC device.

### Device characterization

SEM images were obtained with a Quanta 200 FEG environmental SEM. X-ray diffraction (XRD) patterns were acquired by a Bruker D8 Discover Diffractometer with Cu Kα radiation (1.5406 Å). PL spectrum was measured with a Horiba iHR320 Imaging Spectrometer at room temperature. A 532 nm green laser (Laserglow Technologies) with an intensity of 100 mW cm^−2^ was used as the excitation source. TRPL was obtained using the DeltaPro with a pulsed laser source at 406 nm (Horiba NanoLED 402-LH; pulse width below 200 ps, 20 pJ per pulse, ~1 mm^2^ spot size), and the signal was recorded using time corrected TCSPC. c-AFM measurements were performed on an Asylum Research MFP-3D AFM using Pt-coated Si conductive probes (PPP-EFM, Nanosensors). The conductivity of perovskite films was measured by a two-electrode method on glass substrates. The *J–V* characteristics of the cells (voltage scanning rate 0.1 V s^−1^) and the steady photocurrent under maximum power output bias (0.93 V) were recorded with a Keithley 2400 source-meter under simulated AM 1.5 G irradiation by a Xenon lamp (Oriel 67005), which was calibrated by a silicon diode equipped with a Schott visible color KG5 glass filter. EQE was measured with a Newport QE measurement kit. The *J–V* and EQE measurements were conducted under ambient air conditions without encapsulation. A non-reflective shadow mask was used to define a 0.08 cm^2^ active area of the PSCs. The series resistance (*R*_s_) is derived from the slope of the *J–V* curve at the open-circuit voltage point. TPV decay was measured under 1 sun illumination and recorded by a 1 GHz Agilent digital oscilloscope.

### Data availability

The data that support the findings of this study are available from the corresponding author on reasonable request.

## Electronic supplementary material

Supplementary Information
