# Supplementary material for: Molecular doping enabled scalable blading of efficient hole-transport-layer-free perovskite solar cells
Source: Nat Commun. 2018 Apr 24;9:1625. doi: 10.1038/s41467-018-04028-8 (PMC5915422; doi:10.1038/s41467-018-04028-8)
Supplement: Supplementary file 1 — Supplementary Information [file 41467_2018_4028_MOESM1_ESM.pdf]

Molecular Doping Enabled Scalable Blading of Efficient Hole-Transport-Layer-Free Perovskite Solar Cells

Wu et al.

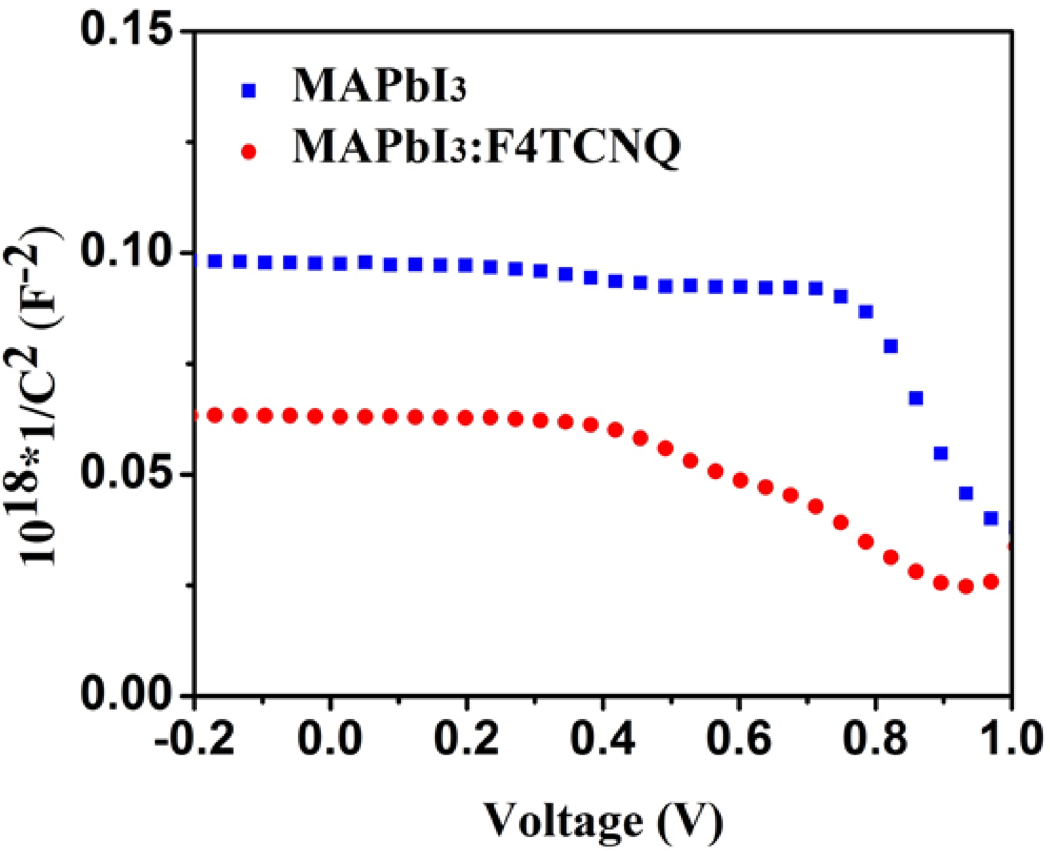

Supplementary Figure 1| Carrier concentration characterization. Mott-Schottky plot (dark capacitance versus voltage) of HTL-free PSCs based on $MAPbI_3$ or $MAPbI_3$:F4TCNQ films.

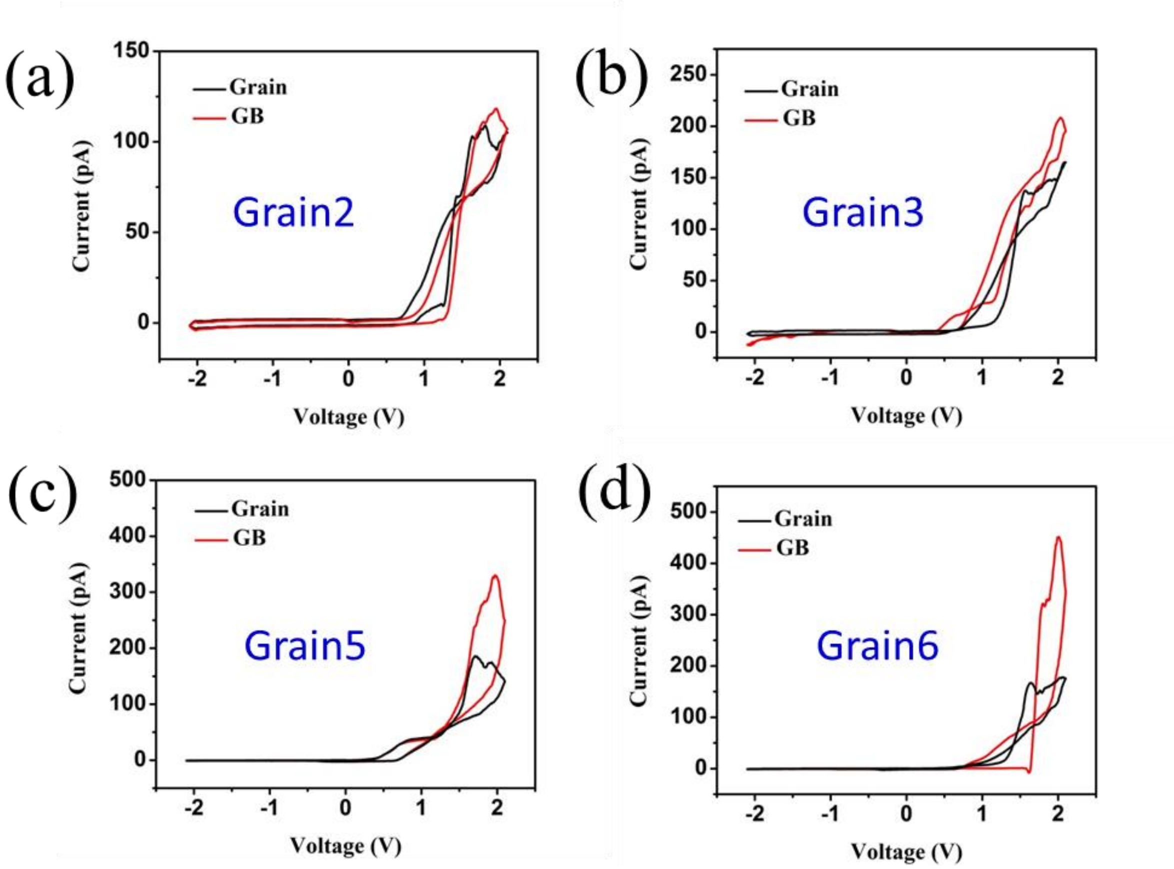

Supplementary Figure 2| Conductivities of neat or doped perovskite films. Local dark current measured at the GBs and on the grains for the (a, b) $MAPbI_3$ film as indicated in Figure 2e, and (c, d) $MAPbI_3$:F4TCNQ film as indicated in Figure 2g.

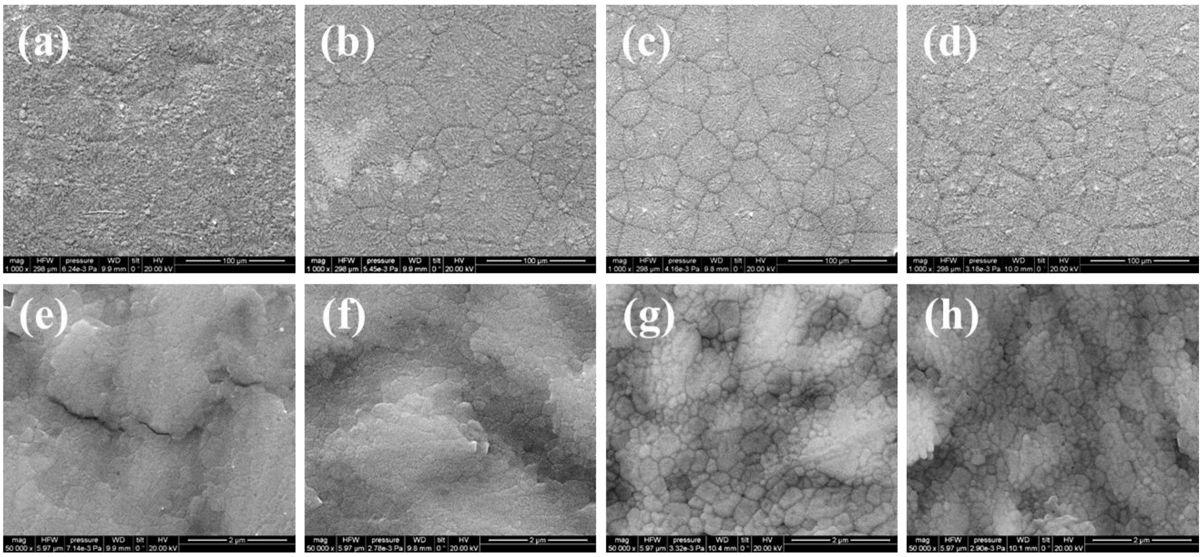

Supplementary Figure 3| F4TCNQ dopant concentration optimization. SEM images of bladed $MAPbI_3$ films prepared with different amounts of F4TCNQ: (a, e) 0.01 wt%, (b, f) 0.02 wt%, (c, g) 0.03 wt%, and (d, h) 0.05 wt%.

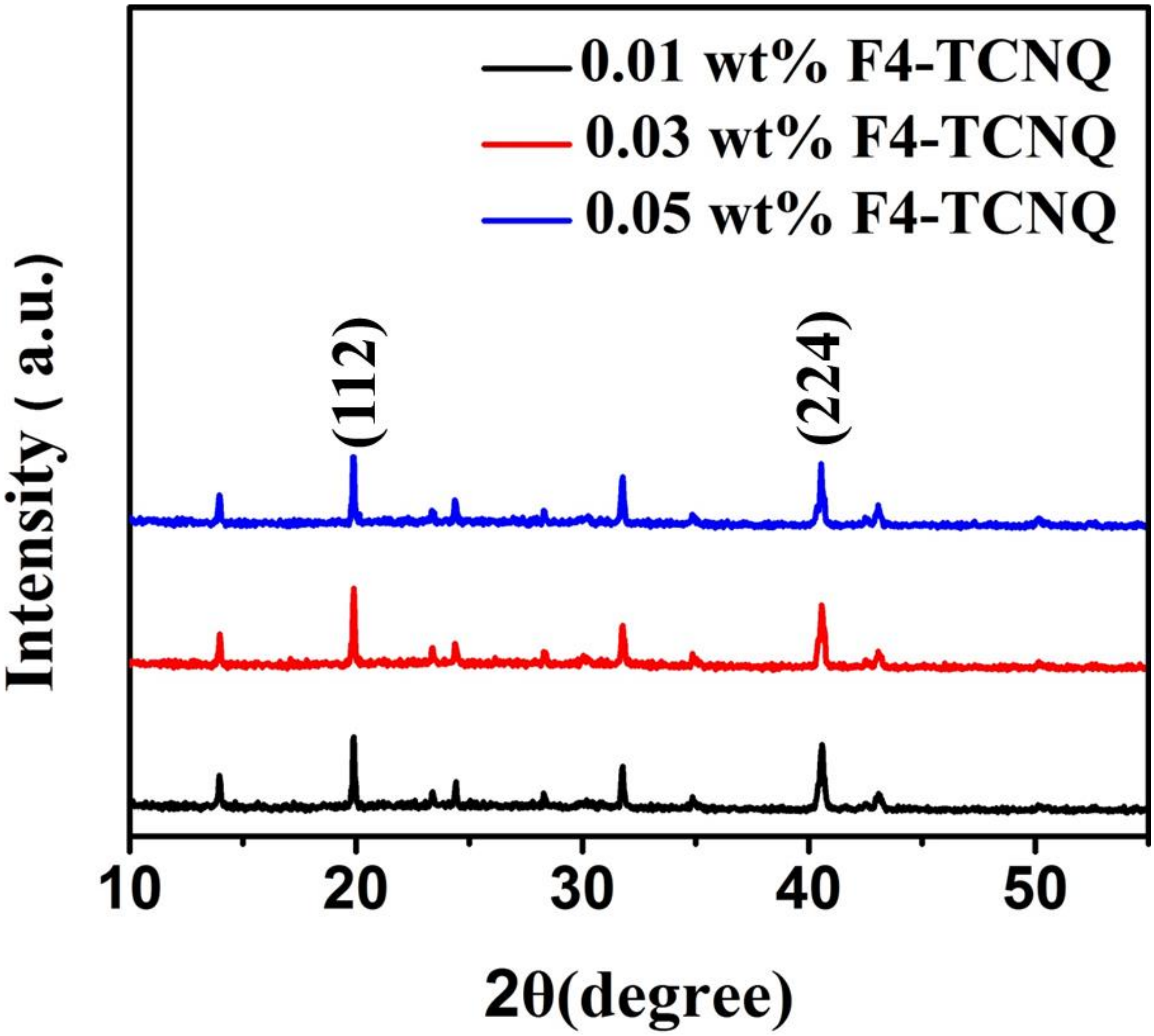

Supplementary Figure 4| XRD characterization based on F4TCNQ dopant concentration optimization. XRD patterns of the bladed $MAPbI_3$ films prepared with different amounts of F4TCNQ. The introduction of F4TCNQ did not affect the crystallinity of the perovskite films.

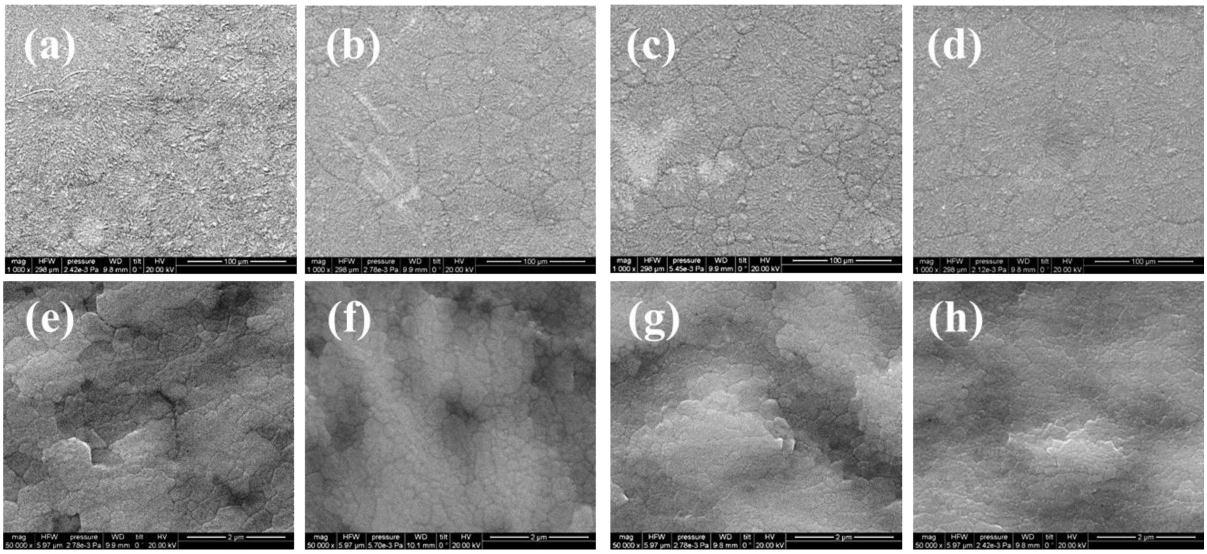

Supplementary Figure 5| MHP additive concentration optimization. SEM images of bladed $MAPbI_3$ films prepared with 0.03 wt% F4TCNQ and different amounts of MHP: (a, e) 0.075 wt%, (b, f) 0.150 wt%, (c, g) 0.225 wt%, and (d, h) 0.300 wt%.

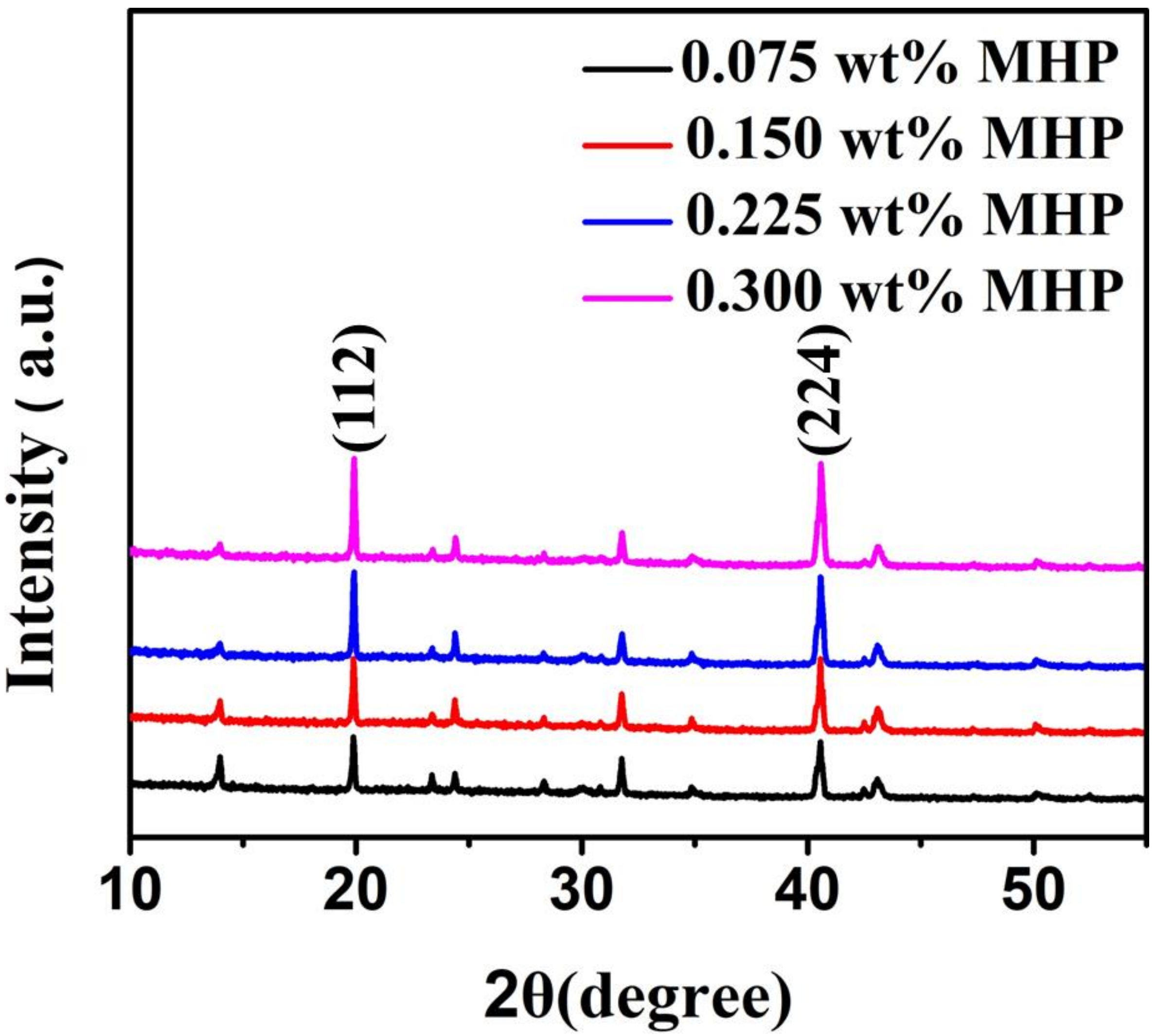

Supplementary Figure 6| XRD characterization based on MHP additive concentration optimization. XRD patterns of the bladed $MAPbI_3$ films prepared with 0.03 wt% F4TCNQ and different amounts of MHP.

Increasing the MHP concentration in the precursor solution led to enlarged grains and enhanced crystallinity of for the perovskite films (Supplementary Fig. 5 and Fig. 6).

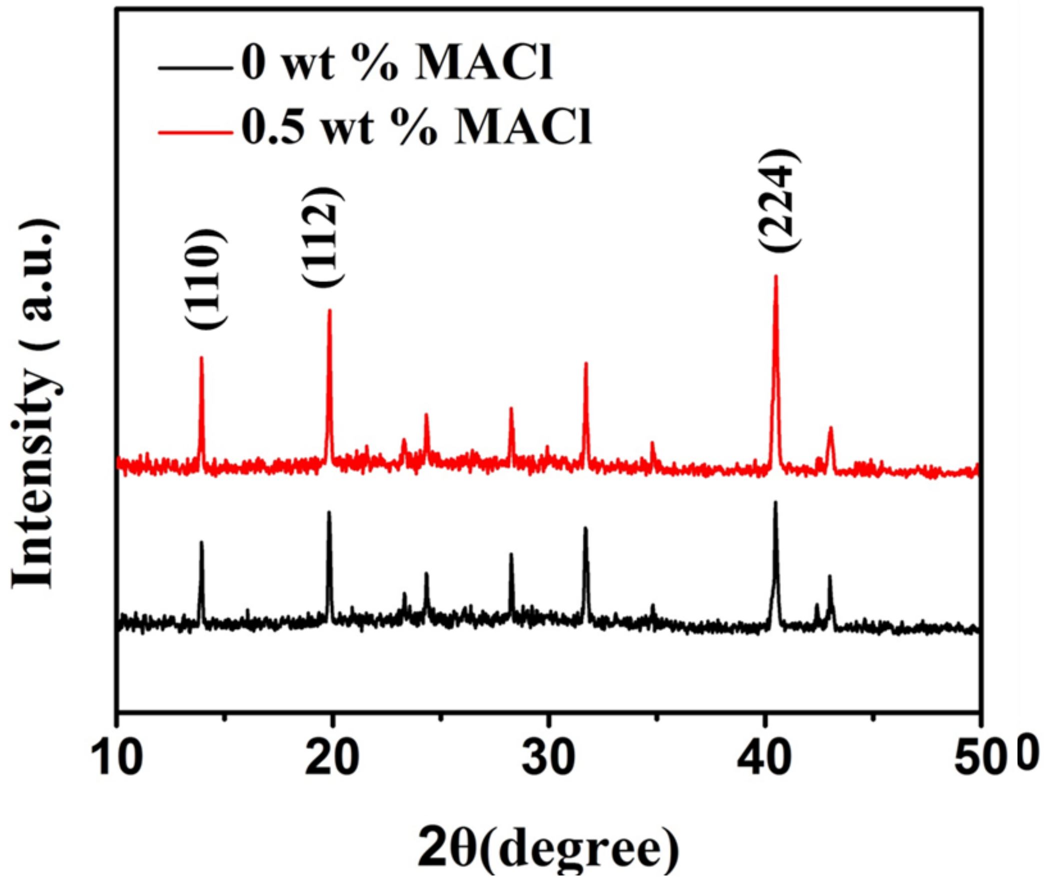

Supplementary Figure 7| XRD patterns of bladed perovskite films prepared using a co-solvent annealing technique in the absence or presence of MACl.

The main $MAPbI_3$ XRD peaks, i.e. (110), (112) and (224), intensified after the introduction of 0.5 wt% excess MACl (Supplementary Fig. 7), suggesting that chlorine plays an important role in improving the crystallinity of perovskite films.

Supplementary Table 1| Photovoltaic parameters obtained from HTL-free PSCs employing bladed $MAPbI_3$ films incorporated with different amounts of F4TCNQ.

**F4TCNQ concentration (wt%)**	J_{sc}/mA cm^{-2}	V_{oc}/mV	η/%	*Average* η/%	**FF**
0.01	21.9	1.08	16.7	14.55±0.50	0.71
0.02	22.2	1.10	18.4	16.78±0.42	0.75
0.03	22.7	1.10	20.2	18.85±0.35	0.81
0.05	22.4	1.08	19.1	17.69±0.38	0.79

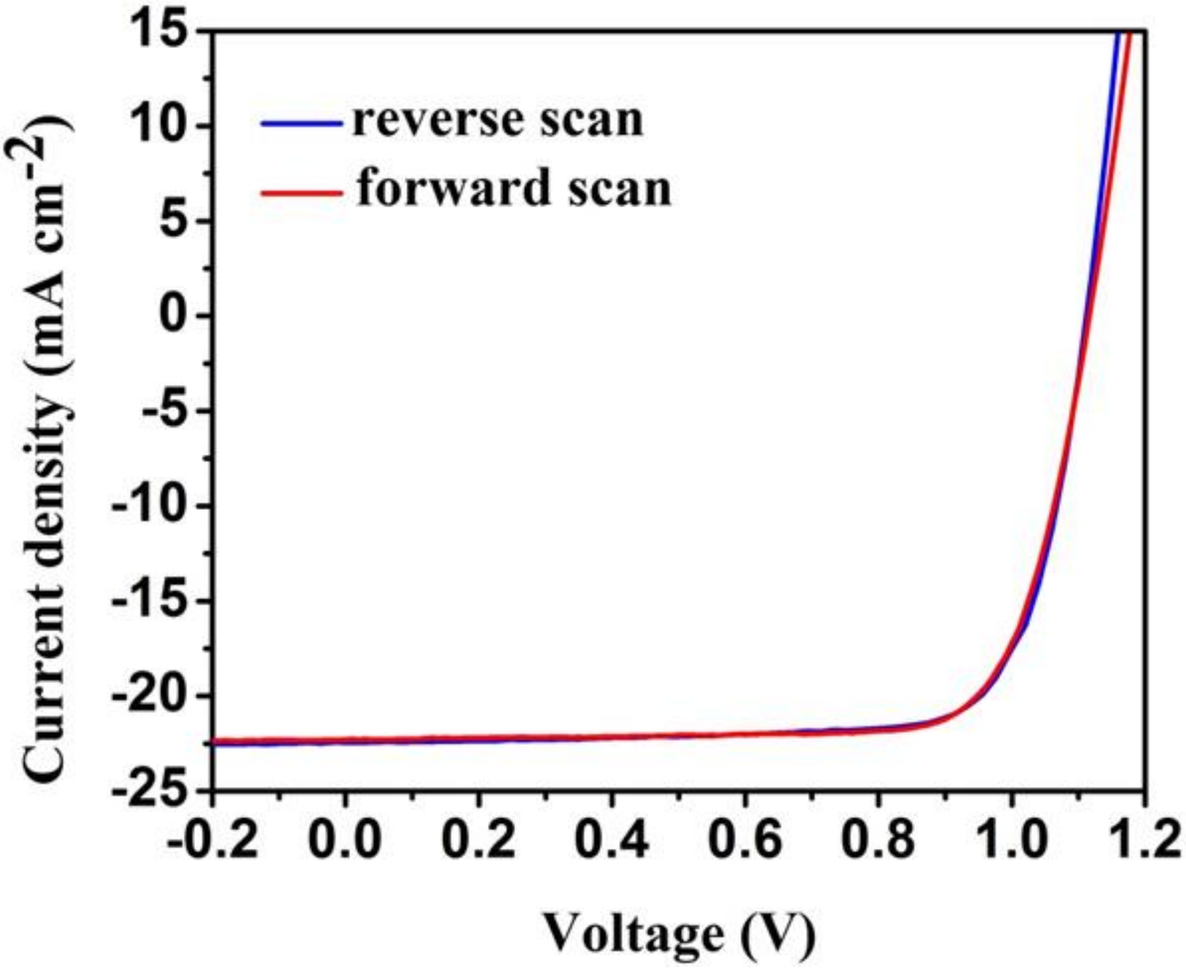

Supplementary Figure 8| *J-V* curves hysterisis. *J-V* characteristics of the PSC based on $MAPbI_3$:F4TCNQ film measured under different scanning directions.

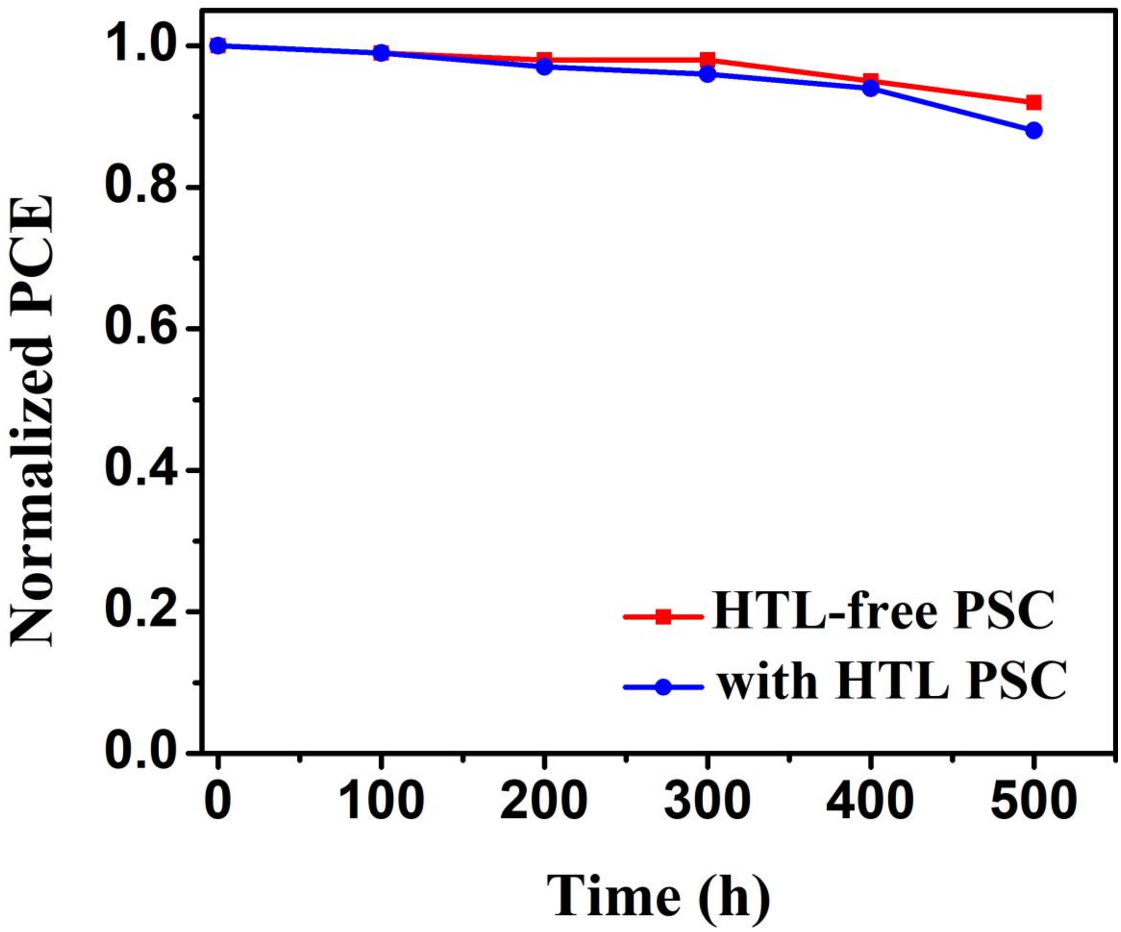

Supplementary Figure 9| Stability test. The stability test as a function of storage time in ambient conditions for PSCs in the absence or presence of HTL.

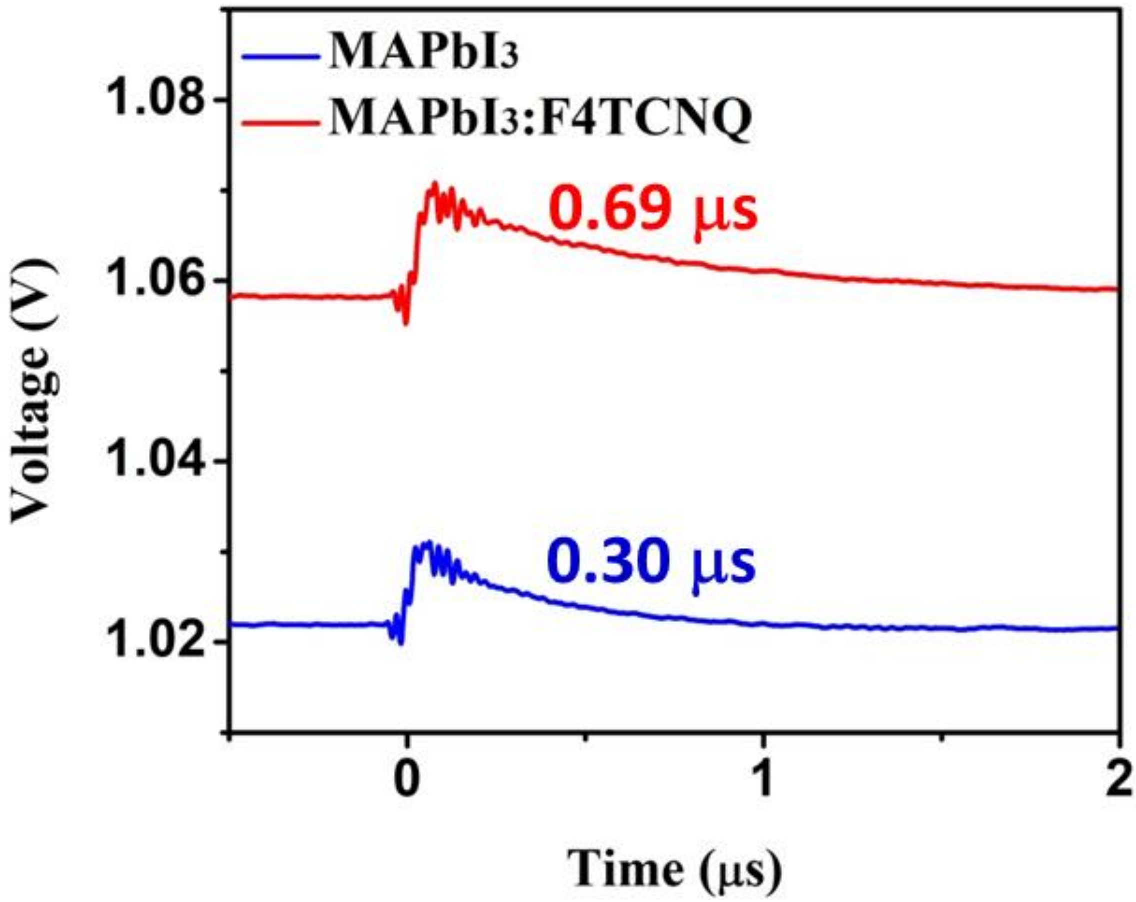

Supplementary Figure 10| Carrier recombination characterization. TPV of HTL-free PSCs based on $MAPbI_3$ or $MAPbI_3$:F4TCNQ films.

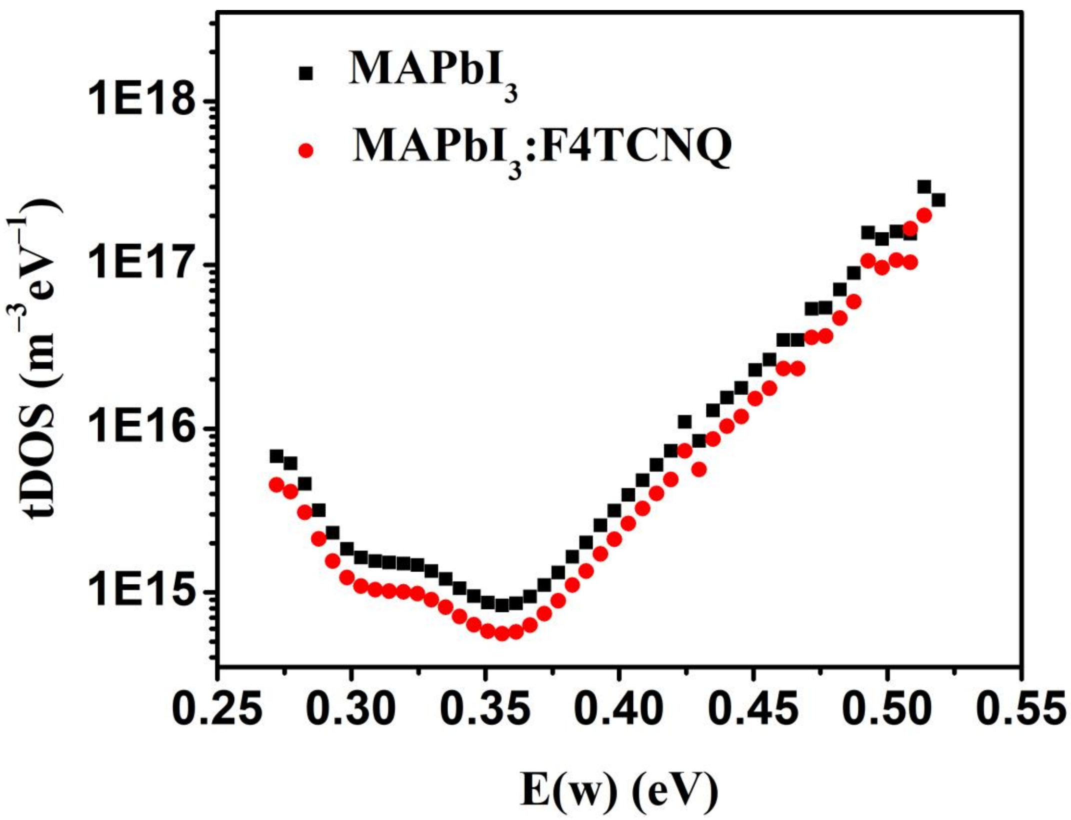

Supplementary Figure 11| Trap density characterization. Trap density of states obtained from TAS measurement for devices based on $MAPbI_3$ and $MAPbI_3$:F4TCNQ.
